# Structural determination of human nucleosomes reconstituted by the ExACT platform

**DOI:** 10.64898/2026.07.30.741638

**Published:** 2026-07-30

**Authors:** Kei-ichi Okimune, Taiki Azuma, Petra Banko, Junko Haga, Genki Terashi, Ryo Morishita, Yaeta Endo, Shunsuke Kita, Daisuke Kihara, Katsumi Maenaka, Taichi E. Takasuka

## Abstract

The structure and function of eukaryotic chromatin have been extensively studied using conventional salt dialysis-based nucleosome assembly, which has provided fundamental structural insights into nucleosomes as well as a mechanistic understanding of their roles in DNA replication, repair and gene expression. Recently, we developed a labor-saving and time-efficient nucleosome assembly method using a wheat germ cell-free Expression and Assembly Coupled Technology (ExACT), which is emerging as a powerful tool for chromatin research. Here we report cryo-electron microscopy (cryo-EM) structures of human H3.1- and H3.3-containing nucleosomes assembled using this approach and validated by deep-learning-based amino acid-wise model quality (DAQ) scoring. The structures of H3.1- and H3.3-nucleosomes are nearly identical to previously reported models, confirming the structural fidelity of the method. In addition, we determined the previously unreported structure of the primate-specific H3.X-containing nucleosome. Together, these findings validate the cell-free co-expression nucleosome assembly platform and establish this method as a robust framework for biochemically investigating chromatin dynamics.

## Full Text Availability

The license terms selected by the author(s) for this preprint version do not permit archiving in PMC. The full text is available from the preprint server.

